# *Streptococcus pyogenes* Lineage ST62/*emm*87: The International Spread of This Potentially Invasive Lineage

**DOI:** 10.3390/antibiotics12101530

**Published:** 2023-10-11

**Authors:** Caroline Lopes Martini, Deborah Nascimento Santos Silva, Alice Slotfeldt Viana, Paul Joseph Planet, Agnes Marie Sá Figueiredo, Bernadete Teixeira Ferreira-Carvalho

**Affiliations:** 1 Departamento de Microbiologia Médica, Universidade Federal do Rio de Janeiro, Rio de Janeiro 21941-902, RJ, Brazil; carol.martini@micro.ufrj.br (C.L.M.); deborah@micro.ufrj.br (D.N.S.S.); alice.viana@micro.ufrj.br (A.S.V.); 2 Perelman School of Medicine, University of Pennsylvania, Philadelphia, PA 19104, USA; planetp@chop.edu; 3 Children’s Hospital of Philadelphia, Philadelphia, PA 19106, USA; 4 Programa de Pós-graduação em Patologia, Faculdade de Medicina, Universidade Federal, Fluminense, Niterói 24220-900, RJ, Brazil

**Keywords:** ST62, *emm*87, *Streptococcus pyogenes*, flesh-eating diseases, scarlet fever

## Abstract

*Streptococcus pyogenes* is known to be associated with a variety of infections, from pharyngitis to necrotizing fasciitis (flesh-eating disease). *S. pyogenes* of the ST62/*emm*87 lineage is recognized as one of the most frequently isolated lineages of invasive infections caused by this bacterium, which may be involved in hospital outbreaks and cluster infections. Despite this, comparative genomic and phylogenomic studies have not yet been carried out for this lineage. Thus, its virulence and antimicrobial susceptibility profiles are mostly unknown, as are the genetic relationships and evolutionary traits involving this lineage. Previously, a strain of *S. pyogenes* ST62/*emm*87 (37–97) was characterized in our lab for its ability to generate antibiotic-persistent cells, and therapeutic failure in severe invasive infections caused by this bacterial species is well-reported in the scientific literature. In this work, we analyzed genomic and phylogenomic characteristics and evaluated the virulence and resistance profiles of ST62/*emm*87 *S. pyogenes* from Brazil and international sources. Here we show that strains that form this lineage (ST62/*emm*87) are internationally spread, involved in invasive outbreaks, and share important virulence profiles with the most common *emm* types of *S. pyogenes*, such as *emm*1, *emm*3, *emm*12, and *emm*69, which are associated with most invasive infections caused by this bacterial species in the USA and Europe. Accordingly, the continued increase of ST62/*emm*87 in severe *S. pyogenes* diseases should not be underestimated.

## 1. Introduction

### 1.1. Streptococcus pyogenes Infections

*Streptococcus pyogenes*, also known as group A *Streptococcus* (GAS), can be associated with various infections, such as pharyngitis, impetigo, cellulitis, and abscesses. This bacterium is an important reemerging pathogen associated with an increasing number of invasive diseases such as septicemia, streptococcal toxic shock syndrome (STSS), and necrotizing fasciitis [1]. Repeated GAS infections may lead to autoimmune sequelae, including rheumatic fever. With the establishment of surveillance programs in many developed countries, data on the incidence and severity of *S. pyogenes* diseases has been accumulating, allowing scientists to have access to important epidemiological data contributing to a better understanding and control of these diseases [2]. In New Zealand, the overall incidence of infections caused by invasive group A *Streptococcus* (iGAS) during a 6-year period was 5.6 per 100,000 (95%CI 4.1–7.4) [3]. In Idaho, USA, from 2008 to 2019, the incidence increased from 1.04 to 4.76 cases/100,000, and two outbreaks were identified [4].

M protein, a surface immunodominant antigen of *S. pyogenes*, is an important determinant of bacterial virulence. This protein is encoded by the *emm* gene, whose hypervariable region is the base for the *emm* typing of *S. pyogenes* strains. GAS can be classified into more than 240 *emm* types and 1000 subtypes, whose distribution patterns can vary regionally [5].

In France, from 2009 to 2017, a total of 61 different types of *emm* were identified, and the most frequent were *emm*28 (16%), *emm*89 (15%), *emm*1 (14%), and *emm*4 (8%), representing >50% of circulating GAS strains. However, other types of *emm* (*emm*44, *emm*66, *emm*75, *emm*83, *emm*87) emerged continually. Furthermore, among emerging types, *emm*75 and *emm*87 showed increased prevalence with persistent annual incidence, demonstrating the risk of clonal expansion [6]. A study on iGAS, conducted from January 2000 to May 2017, in Europe and the USA, demonstrated that the most identified types were *emm*1, *emm*28, *emm*89, *emm*3, *emm*12, *emm*4, and *emm*6, and together they represented approximately 50–70% of total GAS strains in North America. The *emm*1 type prevailed in most surveys conducted in the United States, followed by *emm*12, *emm*28, and *emm*3. However, in the Thunder Bay District of Canada, unusual types predominated, and the most prevalent were *emm*87 (12.3%), *emm*82 (10.8%), *emm*1, *emm*101, and *emm*83 (9.2% each), and *emm*114 (7.7%) [7].

### 1.2. The Increased Emergence of ST62/emm87 Lineage

Some studies have correlated certain types of *emm* with more severe clinical manifestations and disease. Despite the prevalence of specific types of *emm* types in invasive diseases, such as *emm*1, recent isolates of *emm*87 have been associated with family- and hospital-acquired outbreaks of invasive *S. pyogenes* infections. Due to this pattern, it has been proposed that these GAS isolates are highly transmissible [8,9]. In fact, other studies have shown that the prevalence of *emm*87 has increased among iGAS infections in different countries, such as Portugal, Spain, and France [6,10,11], being one of the five most frequent *emm* types in Europe and thirteenth in the USA [7].

Recent studies with an *emm*87 knockout mutant of the *S. pyogenes* strain 20161436, isolated from an invasive disease in Minnesota, USA, showed that the Δ*emm*87 knockout was less cytotoxic to THP-1 macrophage-like cells and that purified M87 showed increased dose-dependent cytotoxicity. Furthermore, mature 1L-1β release was decreased in *Δemm*87-infected macrophages, indicating that M87 can trigger mature 1L-1β release. Additionally, the loss of M87 was found to cause an increase in *S. pyogenes* clearance. The *emm*87 knockout showed reduced survival in whole blood and neutrophil killing assays, although it did not sensitize *S. pyogenes* cells to killing by serum, to which it was intrinsically resistant. Furthermore, infection with the *emm*87 knockout mutant led to a greater neutrophil oxidative burst compared with *S. pyogenes* wild-type (WT) and *emm*87-complemented strains. Finally, in a systemic mouse model, mortality was reduced in the Δ*emm*87 compared with WT and *emm*87-complemented strains. Thus, it has been suggested that *emm*87 is involved in the pathogenicity of GAS by modulating the interaction between *S. pyogenes* and innate immune cells [12].

### 1.3. Therapeutic Failures and the ST62/emm87 Lineage of S. pyogenes

Although GAS remains universally susceptible to penicillin, an important aggravating factor of *S. pyogenes* infections, including iGAS, is therapeutic failure, which can occur even when the appropriate antimicrobial is used to treat such infections. Treatment failure has remained a problem for years and can occur during the treatment of relatively simple illnesses such as pharyngitis to more severe invasive diseases. Treatment failure in invasive infections can have high failure rates. It was estimated in 2005 that the burden of iGAS diseases is unexpectedly high, with at least 663,000 new cases and 163,000 deaths per year [13], and there is no evidence that this rate is decreasing. Several mechanisms have been implicated in therapeutic failures, including the “Eagle” effect, tolerance, decreased penetration of the antibiotic into biofilms, and the development of persister cells [14]. However, the latter seems to be the most accepted mechanism currently to explain failures in different bacterial species to various types of antimicrobials. Persisters are dormant cells that exhibit inhibition of protein synthesis, metabolism, and impairment of cell division so that they do not respond to the action of antimicrobials that require active bacterial replication for action. Recently, using proteomic and other molecular approaches, we demonstrated that the *S. pyogenes* strains 37–97 (ST62/*emm*87) can form persister cells that are refractory to different types of drugs, including β-lactams and clindamycin, indicated for the treatment of iGAS [15]. In addition to impaired cell growth and protein inhibition, persisters of ST62/*emm*87 background also showed an increased efflux capacity that could also be involved in the observed antimicrobial refractoriness.

### 1.4. Study Aims

Despite the increasing number of *S. pyogenes* sequence submissions to the Sequence Read Archive (SRA)—5 in 2009 to 47,936 in August 2023—most strains were from Europe and North America [16,17]. In the GenBank, 2241 genome sequences of *S. pyogenes* from different lineages are available, of which only 270 are completely closed genomes [18]. Of the total of 2241 genomes, only 71 are from Brazil. In addition, to the best of our knowledge, there is no study on the comparative genomics of the ST62/*emm*87 lineage. Thus, to gain insights into the pathogenesis and evolution of this lineage, we performed comparative genomic and phylogenomic analyses using the whole genome sequence (WGS) of the 37–97 strain and other ST62/*emm*87 genomes of international origin, deposited at the National Center for Biotechnology Information (NCBI) databases, such as GenBank and SRA, including those related to severe outbreaks.

## 2. Results and Discussion

Resistance gene analysis using the Comprehensive Antibiotic Resistance Database (CARD) revealed only two resistance genes: *lmrP* and *mefE*. The *lmrP* gene is predicted to encode a major facilitator superfamily (MFS) LmrP protein that has been associated with increased resistance to the antibiotic classes lincosamides, streptogramins, tetracyclines, and macrolides [19]. The *mefE* gene has been reported in other Gram-positive species, such as *Streptococcus pneumoniae*, and plays an important role in macrolide resistance [20]. The analysis of 58 genomes included in this study (Supplementary Materials Table S1) shows the same resistance profile for all ST62/*emm*87 genomes. The exception was the outgroup strain NS6033, which carries the *tetM* gene. These findings demonstrate that strains of the lineage ST62/*emm*87 exhibit no significant variation in terms of antimicrobial resistance compared with strains of other lineages of *S. pyogenes* isolated from various countries. As far as antimicrobial resistance is concerned, *S. pyogenes* has continued to be highly susceptible to almost all classes of antibiotics. Resistance to macrolides (and related compounds) and tetracyclines alone is commonly found among *S. pyogenes* [21].

**Figure 1:**
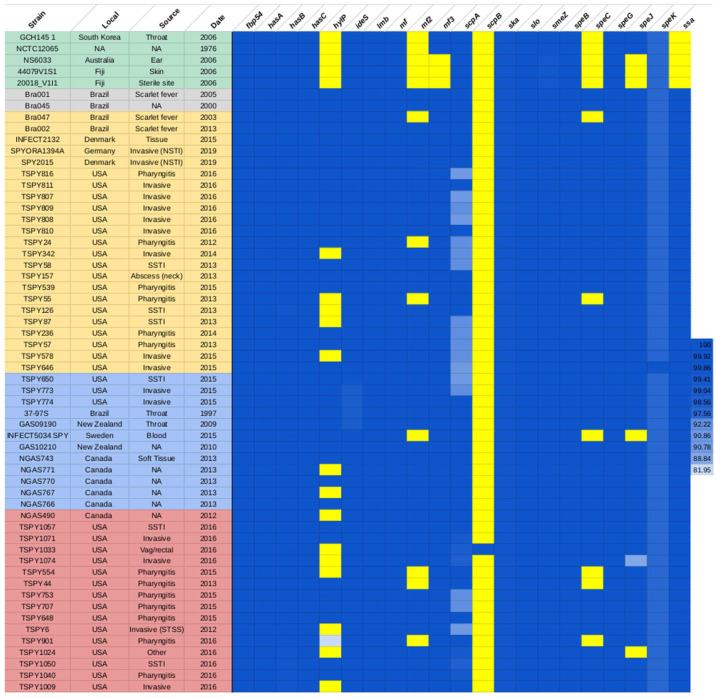
Heatmap of the virulence related-gene profile for the 58 studied genomes of *S. pyogenes* ST62/*emm*87. Strains names are arranged according to the clade structure of the phylogenetic tree shown in Figure 2. Blue rectangle indicates the presence of the gene. The scale indicates the percentage of nucleotide coverage. Yellow rectangle indicates absence of the gene. Percentage of identity data for these genes are presented in Supplementary Materials Table S1. NA: not applicable, SSTI: skin and soft tissue infections, Vag: vagina, NSTI: necrotizing soft tissue infection. STSS: streptococcal toxic shock syndrome.

The analyses of the virulence gene profile of strain 37–97 from Brazil revealed the presence of 20 known virulence-related genes: *fbp54*, which encodes a fibronectin/fibrinogen-binding protein; *hasABC*, hyaluronan synthase A, B, and C; *hylP*, hyaluronoglucosaminidase-phage-associated; *ideS/mac*, Ig protease IdeS domain-containing protein; *lmb*, laminin-binding protein; *mf*/*spd*, streptodornase B; *mf2*, deoxyribonuclease (DNase) Mf2; *mf3*, DNase Mf3; *scpA*, C5a peptidase; *ska*, streptokinase A; *slo*, streptolysin O; *smeZ*, streptococcal mitogenic exotoxin Z; *speB*, streptopain; *speC*, exotoxin type C; *speG*, exotoxin type G; *speJ*, exotoxin type J; *speK*, exotoxin type K; and *ssa*, streptococcal superantigen SSA. Analysis of the other ST62/*emm*87 genomes from NCBI of international origin showed that some virulence genes were highly conserved among these strains and include *hasABC*, *ideS*/*mac*, *lmb*, *mf*/*spd*, *ska*, *slo*, *smeZ*, *speB*, *speG,* and *speK* (Figure 1).

The presence of these various virulence-related genes is consistent with the variety of diseases (from mild to invasive infections and scarlet fever) caused by the *S. pyogenes* of the ST62/*emm*87 lineage (Supplementary Materials Table S1). It is well known that the presence of some virulence factors, especially streptococcal pyrogenic exotoxins (SPEs), is involved in the intense inflammatory response and tissue destruction in *S. pyogenes* severe infections; therefore, characterization of the virulome for specific lineages is of special importance [22,23].

**Figure 2:**
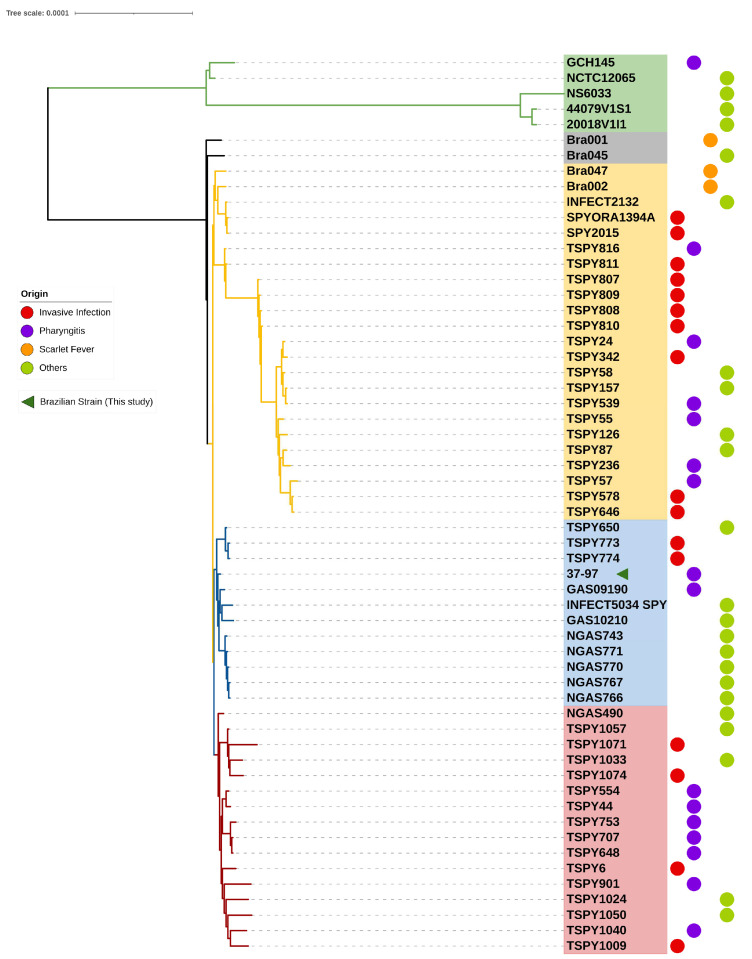
Phylogenetic tree based on core-genome alignment of 58 *S. pyogenes* ST62/*emm*87 genomes. The green arrow shows the location of strain 37–97, a Brazilian strain sequenced by our group that is capable of producing subpopulation of persister cells. The colored circle represents the clinical source.

For the most divergent genomes of strains GCH145, NCTC12065, NS6033, 44079V1S1, and 20018V1I1—which clustered into a completely independent clade and were therefore chosen as outgroups for the phylogenetic tree—the *hylP*, *mf2*, *speC,* and *ssa* genes are absent; however, these four genes were detected in all other ST62/*emm*87 genomes analyzed (Figure 1 and Figure 2). These isolates were recovered mainly from localized infections, except for one that was obtained from a sterile site and one isolate whose clinical site was not reported (Supplementary Materials Table S1).

In the studied collection (*n* = 58), 16 genomes were from invasive diseases that occurred in the USA (*n* = 14), Germany (*n* = 1), and Denmark (*n* = 1). It is important to note that these invasive isolates were distributed in different clades of the phylogenetic tree (Figure 2).

The virulence genes are highly conserved among the analyzed genomes, except for *hylP,* which was lost in the invasive isolates TSPY6, TSPY342, TSPY578, TSPY1009, and TSPY1074, and for polymorphisms found in the *scpA* gene. These data suggest that HylP hyaluronidase is probably not essential for invasive *S. pyogenes* infections. In fact, studies with invasive strains of *S. pyogenes emm*89 from Japan also detected the absence of *hylP* [24].

Of the 50 genomes for which clinical sources were reported, 17 (34.00%) showed higher levels of polymorphism in the *scpA* gene. Of these 17 isolates, a total of 43.75% *(n* = 7/16) were from invasive diseases, while only 29.41% (*n* = 10/34) were from non-invasive diseases (*p* = 0.3181). C5a peptidase (ScpA) is a serine protease that specifically degrades the C5a complement component. It has been suggested that this protease inhibits chemotaxis and may also play a role in host immune evasion [25,26,27]. In *Streptococcus agalactiae*, polymorphisms in *scpB* (*scpA* homolog) can lead to functional differences in protein synthesis [28]. However, the role of *scpA* gene polymorphism in the pathogenesis of *S. pyogenes* diseases needs to be evaluated experimentally.

It is notable that Bra001, Bra002, and Bra047 strains from Brazil were obtained from patients who presented with scarlet fever. These strains shared identical virulence patterns with the Brazilian strain 37–97 (isolated from a pharyngitis case), except that strain Bra047 lacks *mf2* and *speC* genes (Supplementary Materials Table S1). These data seem to indicate that the presence of *mf2* and *speC* may not be critical for the pathogenesis of scarlet fever in this *S. pyogenes* lineage. Corroborating these findings, variation in the content of some genes, including *speC*, was also evident for a global population of scarlet fever strains displaying type *emm*1 [29]. In contrast to the invasive *S. pyogenes*, none of the scarlet fever strains show significant polymorphisms in the *scpA* gene. The *speC* gene was absent in 4 of 13 pharyngitis isolates, the *mef2* gene in 5 isolates, and the *hylP* gene in 2 isolates. Ten isolates were collected from skin/soft tissue infections. Among them, 3 lack the *hylP* gene, and 1 (isolate 44079V1S1) lacks the *hylP* gene and also the *mef2*, *mef3*, *speC*, *speJ,* and *ssa* genes.

In the phylogenetic tree, 51 of the 58 ST62/*emm*87 genomes analyzed were grouped into 3 main clusters, marked in yellow, blue, and red colors (Figure 2). Strain 37–97 from Brazil was grouped in the blue clade, which clustered genomes from EUA, Canada, Sweden, and New Zealand whose strains were collected from 1997 to 2015. The Brazilian strains Bra002 and Bra047 were grouped in the yellow clade; however, the Bra001 and Bra045 strains were in a more basal position outside the main clusters. It is remarkable that strains from all clades were able to cause conditions ranging from uncomplicated pharyngitis to more complicated invasive diseases (Supplementary Materials Table S1 and Figure 2).

When the virulence profile of strain 37–97 was compared with hypervirulent strains of *S. pyogenes* from the USA and Canada [30,31,32,33] that exhibited different types of *emm* and multilocus sequence typing (MLST) (strains M1T15448, ST28/*emm*1; 1838, ST15/*emm*3; MGAS9429, ST36/*emm*12; and MGAS15252, ST172/*emm*59), we found that *fbp*54, *hasABC*, *ideS*/*mac*, *lmb*, *mf*/*spd*, *scpA*, *ska*, *slo*, *smeZ*, *speB*, and *speG* genes were the only virulence-related genes present concomitantly among these hypervirulent strains, and all these genes were present in the strain 37–97 from Brazil. As previously suggested, other factors, including host genetics, may be important for the establishment of invasive diseases. However, more studies are necessary to test this hypothesis [34,35]. In addition, minor genomic variations may contribute to bacterial hypervirulence.

It has been observed that some specific mutations can discriminate between GAS strains isolated from invasive and non-invasive diseases. A mutation that inactivates the two-component virulence repressor system CovRS may facilitate a more severe disease caused by GAS [8,36,37]. For example, the genomes of the ST62/*emm*87 isolates, namely TSPY807, TSPY808, TSPY809, TSPY810, TSPY811, and TSPY816, were obtained from GAS isolates that caused a fatal intrafamily cluster of severe invasive disease in four siblings. An 8-bp duplication (insertion) was previously detected in the CovS-encoding gene of these TSPY genomes, resulting in a reading frame shift and a premature stop codon at position 28 compared with wild-type *covS* [8]. Because CovR suppresses the production of the antiphagocytic hyaluronic acid capsule, high-level production of the capsule is likely essential for the hypervirulent phenotype induced by CovRS inactivation [8,37].

A study by Galloway-Peña and colleagues [38] examined the effects of CovRS inactivation on acapsular strains of serotype M4. Two strains were used: a wild-type strain (M4-SC-1) and a naturally occurring CovS-inactivated strain (M4-LC-1) that contains an 11-bp *covS* insertion. Strain M4-LC-1 exhibited increased expression of surface proteins belonging to the Mga regulon, since Mga inactivation led to the reversal of this effect. Furthermore, only the M4-LC-1 strain showed upregulation of these surface proteins and several others, while the M4-SC-1 strain did not. Notably, the M4-LC-1 strain was more virulent in a mouse model of bacteremia, despite inducing fewer skin lesions in the same model. Thus, the findings demonstrate the importance of the *covS* inactivation mechanism in the virulence of *S. pyogenes*, shedding light on the mechanisms of hypervirulence and pathogenicity of this bacterium [38]. It is important to mention that in addition to the six strains TSPY807-811 and TSPY816 mentioned previously [8], we found in the collection studied here two other GAS strains (INFECT5034_SPY and Bra047) that have the *covS* gene inactivated. The natural *covS* mutation in Bra047 (isolated from a case of scarlet fever in Brazil) was due to an 11-bp insertion (5′ AGAAAATGCAG 3′) in this gene, and INFECT5034_SPY from Sweden (isolated from blood) had exactly the same 8-bp insertion (5′ CTTTTTTT 3′) found in strains TSPY807-811 and TSPY816.

Previous studies using different lineages of GAS strains implicated in streptococcal toxic shock syndrome (STSS) indicated differences in the ST62/*emm*87 strains with respect to gene content (presence of *speK*) and genetic polymorphisms for *dpiB*, which encodes a positive regulator of *maeE* encoding the malic enzyme. However, the exact role played by each genetic variation, in conjunction with the host response, in the pathogenesis of STSS and other severe infections caused by GAS remains unclear [39]. In our analysis using NGAS743 as a reference, all ST62/*emm*87 genomes analyzed from invasive and non-invasive GAS carried the *speK* gene with 100% identity and coverage, except GCH145_1, which showed 99.86% identity and 100% coverage. Regarding *dpiB* polymorphism, only TSPY6, TSPY57-58, TSPY157, TSPY539, TSPY578, TSPY646, SPY2015, and SPYORA1394A showed variability. Thus, only 3 of the 16 ST62/*emm*87 strains classified as iGAS present this polymorphism. Consequently, we were unable to associate the presence of the *dpiB* polymorphism with the severity of the infections.

Strain 37–97 from Brazil presented four regions consistent with known complete bacteriophages: PHAGE_Strept_315.3_NC_004586 (41), PHAGE_Strep_315.2_NC_004585 (18), PHAGE_Strep_315.4_NC_004587 (32), and PHAGE_Strep_315.4_NC _004 587 (36). The average GC content of each was 38.16%, 37.25%, 39.08%, 38.59%, and all *int* genes were confirmed via UNIPROT blast submission (http://www.uniprot.org; accessed on 10 July 2023) and showed 100% identity. The genomic organization of these phages includes genes dedicated to regulation, DNA replication, DNA restriction, genome packaging, phage morphology, and cell lysis. These phages also carried virulence-associated genes such as hyaluronidase (UNIPROT: A0A4D6BC74), C5a peptidase (UNIPROT: Q99Z24), and superantigen A (UNIPROT: A0A4D6AEG5).

The distribution of these phages in the genomes of the other 57 GAS analyzed was variable. Only 13 of the 57 genomes carried these four bacteriophages. Although most of these genomes (carrying these four bacteriophages) were grouped in the yellow clade together with the Brazilian GAS 37–97 (*n* = 8/14). Genomes carrying these four phages were also found distributed in all clades, except in the outgroups. A total of 30 genomes grouped into different clades contained at least two of these bacteriophages [PHAGE_Strep_315.2_NC_004585 (18) and PHAGE_Strept_315.3_NC_004586 (41)]. Taken together, these data suggest a horizontal and promiscuous dissemination of these two phages among ST62/*emm*87 strains. Phage-related genes may constitute approximately 50% of the accessory genes of *S. pyogenes* [16]. However, despite playing a key role in the evolution of *S. pyogenes* lineages and virulence diversity, their role in the genomic variability of *S. pyogenes* is still incompletely understood [40].

The main limitation of this study is related to the small number of genomes analyzed. This is due to the fact that genomes from invasive and serious GAS infections are rare and that relatively few strains of this lineage cause invasive infections in most countries. Furthermore, most isolates are from the USA and are related to outbreaks; thus, there is a low diversity of strains sequenced. Additionally, the data obtained from the NCBI BioSample regarding the type of infection is limited and sometimes incomplete, which may have influenced the definition of invasive and non-invasive infections.

Severe infections caused by *S. pyogenes* are relatively rare compared with mild to moderate infections, but they are associated with a high mortality rate and may increase in prevalence. The lineage of GAS may be critically important for its ability to cause disease. *S. pyogenes* strains of the ST62/*emm*87 lineage are spread in different countries as agents of pharyngitis, scarlet fever, skin/soft tissue infections (SSTI), streptococcal toxic shock syndrome (STSS), and invasive diseases, with increasing frequency and ability to cause outbreaks/infection clusters. Its potential to cause highly lethal invasive diseases and to develop mutations (such as those occurring in *covS*) that can lead to hypervirulence is a cause for concern. Importantly, these strains share many of the known virulence-related genes, including those frequently detected in hypervirulent invasive strains. It is possible for a bacterial strain to transition from a less virulent form to a more virulent form. However, the genetic mechanisms that lead to strains of *S. pyogenes* within the same lineage causing everything from simple pharyngitis to toxic shock syndrome and necrotizing fasciitis remain to be better elucidated. The possibility of the generation of persisters by isolates of this lineage as a potential cause of therapeutic failure is an additional threat, especially in serious invasive diseases including STSS and flesh-eating diseases [15].

## 3. Materials and Methods

### 3.1. Comparative Genomics

We performed a retrospective genomic study of *S. pyogenes* strains of the ST62/*emm*87 lineage. We selected all 16 assembled and annotated ST62/*emm*87 genomes deposited in GenBank, which were downloaded from the Genome Tree Report (https://ncbi.nlm.nih.gov/tools/treeviewer/*;* accessed on 15 April 2022) using the completely closed genomes of the NGAS743 strain (ST62/*emm*87) as reference. Additionally, we downloaded other genomes from the SRA database that were selected based on previous studies [8] and to cover different countries. A total of 41 genome reads (fastq) deposited in the NCBI Sequence Read Archive—SRA files (https://www.ncbi.nlm.nih.gov/sra; accessed on 15 April 2022) were downloaded and the adapter sequences and low-quality sequences were trimmed with TrimGalore version 0.6.4 (https://github.com/FelixKrueger/TrimGalore; accessed on 17 April 2022). The assembly was performed using Spades incorporated into the PATRIC platform (https://www.patricbrc.org/; accessed on 17 April 2022). The genome of the *S. pyogenes* strain 37–97 (GenBank accession: 37–97S; NZ_CP041408.1), sequenced by us [15], was annotated using RAST (Rapid Annotation Using Subsystem Technology) [41]. Previously, 37–97 genome sequence was trimmed using BBDuk Trimmer (version 1.0; accessed on 14 January 2019), and genome assembly was carried out using Newbler v3.0 [42]. Scaffolds were aligned against a reference genome (*S. pyogenes* strain NGAS743; GenBank Accession: CP007560) using Cross Match (version 0.990329; http://www.phrap.org/phrap.docs/phrap.html, accessed on 10 February 2019). Intra-scaffold and inter-scaffold gaps resulting from repetitive sequences were resolved by in silico gap filling [15].

The clinical source reported in this study was obtained from the NCBI BioSample. The genomes of *S. pyogenes* strains obtained from necrotizing fasciitis (SPYORA1394A and SPY2015) and streptococcal toxic shock syndrome (STSS) (TSPY58) were included in the invasive infection group. One isolate obtained from blood and another collected from a sterile site were not incorporated into the group of invasive infection because there was no accurate information about the clinical condition of the correspondent patients.

Multilocus sequence type (MLST) was performed using genome sequences submitted to MLST 2.0, hosted at the Center for Genome Epidemiology (CGE) (https://cge.cbs.dtu.dk/services/MLST/; accessed on 1 May 2022). For functional annotation of the total 58 analyzed genome sequences, the software ABRicate 0.9.8 (https://github.com/tseemann/abricate; accessed on 1 May 2022) was used with the following databases: antimicrobial resistance (Resfinder and CARD) and virulence (VFDB) genes.

Whenever necessary, manual curation was performed using UniProt (http://www.uniprot.org/; accessed on 5 May 2022) and/or the NCBI (https://blast.ncbi.nlm.nih.gov/Blast.cgi; accessed on 5 May 2022) databases. Additionally, the Map to Reference tool on the Geneious Prime platform version 2023.2.1 (Biomatters Inc.; Boston, MA, USA) and the BLAST command line (https://www.ncbi.nlm.nih.gov/books/NBK569856/; accessed on 5 September 2023) were used for further comparisons of genetic sequences grouped in a given phylogenetic cluster.

Flores et al. [8] described a mutation (8-pb insertion) in the *covS* response regulator from the two-component regulator CovRS in ST62/*emm*87 isolates, which may possibly increase the virulence potential of *S. pyogenes*. To test the presence of this insertion in other ST62/*emm*87 genomes analyzed here, the mutated sequence of the *covS* gene from TSPY809 (SRA accession: SRS1935365) and the *covS* sequence from MGAS5005 (reference genome used by Flores et al. [8]; GenBank accession: NC_007297; *covS* locus_tag: M5005_Spy0283) were used. Initially, *covS* sequences were searched in the studied genomes using the BLASTn command line, considering 100% identity and query coverage. Genomes that did not match were analyzed manually using Map to Reference on the Geneious Prime platform version 2023.2.1.

Additionally, Deniskin et al. [39] revealed a polymorphism in the *dpiB* gene (R170C mutation in protein sequence) and unique gene content (*speK*) among *S. pyogenes* ST62/*emm*87 strains involved in pediatric streptococcal toxic shock syndrome and suggested a possible contribution of these genetic profiles in the development of this syndrome by strains of this lineage. Thus, a strategy similar to that used to analyze the *covS* gene was carried out to investigate the *dpiB* polymorphism, but using the *dpiB* gene from the MGAS10750 genome as a reference (GenBank accession: NC_008024). Next, the *dpiB* gene sequence was aligned and compared using the Map to Reference tool on the Geneious Prime platform version 2023.2.1 with that of strain NGAS743 (Genbank accession: CP007560), which belongs to ST62/*emm*87, and this sequence was used as a reference to analyze the 58 genomes studied here. The searches were carried out using the BLASTn command line, with a threshold of 100% identity and coverage. Again, genomes that did not match 100% identity and query coverage were manually inspected using Map to Reference on the Geneious Prime platform version 2023.2.1.

The software Phaster (https://phaster.ca/; accessed on 29 May 2023) was used to search for bacteriophage content in the strain 37–97 genome, and only complete phages were considered in this analysis. The phages sequences found in the 37–97 genome were annotated using Geneious Prime software platform version 2023.2.1 to assess the presence of important genes, such as virulence-associated genes. Additional, sequences of the phages found in 37–97 genome were searched in the other 57 ST62/*emm*87 genomes of this study using the BLAST command line application (https://www.ncbi.nlm.nih.gov/books/NBK569856/; accessed on 20 September 2023).

### 3.2. Phylogenetic Tree

The tree was constructed with 58 *S. pyogenes* ST62/*emm*87 genome sequences available in the GenBank and SRA. These sequences were aligned using ROARY (https://github.com/sanger-pathogens/Roary; accessed on 23 February 2023) and the command “roary -e --mafft -p 8 *.gff” for core genome alignment. The maximum likelihood tree was achieved by employing RaxMLGUI [43] with the GAMMA distribution and GTR substitution model and default parameters with 1000 bootstrap replicates. The tool “Interactive Tree of Life” (iTOL) v.4 was used for tree visualizations and editions [44].

## Data Availability

All data used in this work were reported in the Section 2, Figures, and Supplementary File. Additional information is available on request from the corresponding authors.

## Supplementary Materials

The following supporting information can be downloaded at: https://www.mdpi.com/article/10.3390/antibiotics12101530/s1. Supplementary Table S1: NCBI access number, country, clinical origin, genotype, and antimicrobial and virulence profiles of the 58 studied genomes of the *S. pyogenes* ST62/*emm*87.
